# Maternal mental health and infant and young child undernutrition: A systematic review and meta-analysis

**DOI:** 10.3126/nje.v16i2.95973

**Published:** 2026-08-03

**Authors:** Manisha Singh, John Stephenson, Tomasina Stacey, Julie Abayomi, Sara Eastburn, Padam Simkhada

**Affiliations:** 1 School of Business and Health Studies, York St John University, London, UK; 2 Department of Allied Health Professions, Sport & Exercise, School of Human and Health Sciences, University of Huddersfield, UK; 3 Nursing, Midwifery and Palliative Care, Division of Methodologies, King’s College London, UK; 4 Department of Applied Health and Social Care, Edge Hill University, Liverpool, UK; 5 Research and Innovation Office, University of Chester, UK

**Keywords:** Maternal mental health, child undernutrition, stunting, wasting, underweight

## Abstract

**Background:**

This systematic review and meta-analysis examine the globally available evidence for an association between maternal mental health, such as depression, anxiety and common mental disorders and child undernutrition; both of which are global public health problems.

**Methods:**

Databases including MEDLINE, PsycINFO, CINAHL, Cochrane Library, Global Health Library and relevant reports from the World Health Organisation, United Nations of Children Education Fund and other organisations working in maternal and child health from 1995-2025 were searched. The risk of bias was assessed by recognised tool for assessing cohort studies. Random effects meta-analyses were conducted for all outcomes.

**Results:**

Meta-analysis of the primary outcome revealed that a synthesised estimate of the adjusted odds ratio for the proportion of stunted children of mothers with depression or mental health disorder compared to the proportion of stunted children of mothers with no depression or mental health disorder was 1.41 (95% CI 1.17, 1.69). Secondary meta-analyses revealed significant detrimental effects of maternal depression or mental health issues on underweight. Subgroup analyses suggest that the association is strongest in children up to 2 years of age in middle-income countries, and that there is insufficient evidence for an association in a meta-analysis conducted on low risk-of-bias studies only.

**Conclusion:**

The review provides evidence of a significant association between maternal mental health and child growth disorder.

## Introduction

In recent years, there has been increased awareness of the extent of maternal mental health problems, such as depression and anxiety, and has been identified by the World Health Organisation (WHO) as a major public health issue [1,2]. Nearly 1 in 5 women globally experience some form of mental health condition during pregnancy or after childbirth [2]. In LMICs, depression and anxiety in women during pregnancy and the postnatal period are higher compared to high-income countries (HICs) [3]. Among women with perinatal mental health conditions, 20% are reported to experience suicidal thoughts or act on self-harm [2]. Globally, depression is recognized as the leading cause of disease-related disability in women of childbearing age [4,5]. It is thought to be responsible for reduced maternal response to a child’s needs [3,6].

The independent consequences of maternal mental health issues for women include heightened risks due to various factors associated with childbearing, such as hormonal changes, social pressures, and economic challenges [1,2]. These conditions can significantly impact a woman's quality of life, productivity, and overall health [4,5]. Recognising the importance of maternal mental health in its own right is essential. While this review acknowledges these independent consequences, it primarily focuses on the outcomes for children to highlight the broader implications of maternal mental health on the next generation.

Malnutrition remains a global public health problem, with an estimated 45% of child deaths attributed to undernutrition worldwide [7]. The WHO estimated that globally, 149 million children under 5 years were stunted (low height-for-age), and 45 million were wasted (low weight-for-height) in 2020, with most cases in low- and middle-income countries (LMICs) [8]. Stunting serves as a key indicator of child health and nutritional status [9]. Good nutrition during the first 1000 days of life from conception to a child’s second birthday is a critical period for growth and development. Poor childhood nutritional status can result in inadequate physical growth, poor cognitive development, and reduced economic productivity [10-12]. Early childhood is a period when children are most dependent on their caregivers to meet their nutritional needs [13,14]. Consequently, the health and mental status of primary caregivers, often mothers, significantly influence the child’s wellbeing [15,16].

Several studies have examined the association between maternal mental health and child nutritional outcomes: maternal depression, in particular, has been recognised as a major contributing factor to the poor development of infants and young children [5,17,18]. Evidence suggests that addressing depression in mothers can lead to improvements in children’s growth, and development, and reduce occurrences of childhood diarrhoea and undernutrition [8]. Early detection and treatment of maternal mental health issues are crucial for preventing morbidity and mortality in young children.

While existing reviews have demonstrated an association between maternal mental health and child undernutrition, most focus primarily on maternal depression [11,19,20]. Therefore, this study intends to provide a global perspective on broader maternal mental health issues including depression, anxiety and common mental disorders, and their relationship with undernutrition in children under five years.

## Methodology

The current review was registered on PROSPERO, CRD42020189315. The method used in this review is based on the published protocol for this systematic review [21].

### Ethical considerations

No ethical approval was required for this current study, as it used secondary data collection and analysis.

### Eligibility Criteria

Any observational studies (including cross-sectional, longitudinal, case-control and cohort studies) that investigated an association between maternal mental health and child malnutrition/undernutrition were included in the study.

Studies that examined common mental disorders, such as anxiety and depressive disorders using standardised screening or diagnostic tools, studies that used the WHO standard measurement based on weight for height assessment, height for age, stunting or wasting or mid-upper arm to assess the child’s nutritional status were included in the review. Inclusion criteria were based on the PECO criteria (Population, Exposure, Comparator and Outcome) (Table 1).

### Search strategy

The literature search started with two key phrases: ‘maternal mental health’ and ‘child malnutrition’. Keywords used to search for articles included “child* undernutrition”, “child* malnutrition”, “stunting”, “wasting”, “Low birth weight”, “SAM” (Severe Acute Malnutrition), “MAM” (Moderate Acute Malnutrition), “PEM”, “Protein-Energy- Malnutrition”, “Protein Energy Malnutrition”, “maternal mental health”, “maternal depression”, “depression”, “Maternal anxiety”, “anxiety”, “maternal mental disorder”, “mental disorder”. A subheading search was conducted to identify important studies and ensure literature saturation. Furthermore, bibliographies of those papers that matched the eligibility criteria were searched to identify any further relevant articles.

Articles for this review were sourced from the following electronic databases: MEDLINE (via the PubMed interface), PsycINFO, CINAHL, and Cochrane Library. In addition, Global Health Library Relevant reports from the WHO, United Nations of Children Education Fund (UNICEF) and other organisations working in maternal and child health were also assessed. Database of systematic reviews (CDSR) and database of abstracts of reviews of effects (DARE) were also searched for relevant literature. The search was limited to the literature available in English only. The search for the literature covered the time period from 1995 to December 2025.

### Study Selection

All records identified through the database searches were imported into Mendeley Reference Manager and duplicates were removed. Study selection was conducted in two stages. First, two reviewers (MS and JA) independently screened titles and abstracts to identify potential eligible studies. Second, full-text articles were assessed independently by three reviewers (TS, SE, and PS) against the predefined eligibility criteria. Any disagreements at either screening stages were resolved through discussion, and where consensus could not be reached a fourth reviewer (MS or JA) supported with decision making. This process helped minimise selection bias and ensured methodological rigour.

### Data extraction

Data extraction was conducted independently by three reviewers (MS, JA, JS) using a predefined extraction template. The following information was collected from each included study: study characteristics (author, year, country, study design), sample size, participant characteristics, type of MMH disorder, MMH screening tool, prevalence of MMH, child nutrition outcomes, reported effect estimates such as Odds ratios or mean differences. Any discrepancies in extracted data were resolved through discussion among the reviewers.

### Risk of Bias assessment

The quality of the included studies was evaluated by multiple authors using the Newcastle Ottawa Scale (NOS) [22]. This scale scores each paper on Selection, Comparability and Outcome; with a total of 9 points being available over the three domains; higher scores indicate lower risk of bias. Notably a score of 7 points or more represents a study of high quality, with low risk of bias [23,24].

### Meta-analysis methods

Selected studies were grouped for synthesis based on outcome type and measurement scale. Binary outcomes included stunting, wasting and underweight using WHO cut-offs. Continuous outcomes included HAZ, WHZ and WAZ scores.

Meta-analyses were proposed on the primary outcome of stunting (expressed as a binary outcome); and the secondary outcomes of standardised stunting measure (HAZ); wasting or moderate/severe malnutrition (expressed as a binary outcome); standardised wasting or moderate/severe malnutrition measure (WHZ); underweight (expressed a binary outcome); standardised underweight measure (WAZ).

Random effects meta-analyses were constructed for each outcome using restricted maximum likelihood estimation methods. Random effects models, yielding conservative estimates compared to models based on fixed estimation effects, were chosen to reflect recognised clinical and methodological heterogeneity across included studies with respect to all outcomes.

For analyses of binary measures, synthesised estimates were based on adjusted odds ratios (odds of the event in mothers with depression or mental disorder compared with those in mothers with no depression or mental disorder) and associated 95% confidence intervals (CIs). Where not reported directly, these statistics were calculated from reported events and non-events in both groups. For analysis of z-scores (interval-level numerical measures), synthesised estimates were based on standardised mean differences (SMDs), based on scores reported by mothers with depression minus scores reported by mothers with no depression, and associated 95% CIs. Where not reported directly, standard deviations used in these measures were calculated from reported standard errors or confidence intervals. The main analysis was based on adjusted estimates of effect. To assess the effect of expected variation in the levels of control in each included study, the main findings were compared against those from an analysis based on unadjusted estimates.

Meta-analysis reporting of the primary outcome was via the construction of a forest plot, reporting synthesised estimates and associated 95% CIs, a Z-test for the estimated effect and heterogeneity statistics; including results from Cochran’s Q test for heterogeneity, the I2 statistic (proportion of variation across studies ascribed to heterogeneity) and the τ2 statistic (an estimate of between-study variance). Heterogeneity in the meta-analysis of the primary outcome was further explored with a Galbraith plot. A sensitivity analysis was conducted on the meta-analysis of the primary outcome to assess the robustness of the derived estimates via an influence plot. Small study effect-related bias (including publication bias) was assessed via a funnel plot for the meta-analyses of a primary outcome, subject to a minimum of 10 studies being included in the analysis

Subgroup analyses were initially proposed on the primary outcome based on risk of bias status (including studies assessed by the Newcastle-Ottawa scale as being of high quality at and low risk of bias only); country status (comparing studies conducted in middle-income countries with studies conducted in low-income countries, using World Bank designations of country status); and participant type (comparing boys versus girls; and comparing children aged 0-6 months, children aged 6-24 months, and children aged 25-60 months). None of the studies reported results separately for boys and girls, including either stunting outcome, precluding proposed subgroup analysis of the sex of the child. Subgroup analyses based on the age variable were subsequently re-defined to compare children aged 0-6 months and children aged 0-24 months. This was due to many included studies analysing children whose age range spanned multiple age groups defined by the proposed subgroup analysis based on the child’s age.

Synthesised effects from sub-groups and all secondary analyses were reported in tabulated form. All significance testing was conducted at the 5% level of significance.

## Results

The initial search yielded 1427 articles: 833 after removing duplicates. These were screened by title and abstract independently by two authors (MS and JA) to identify those that potentially met the inclusion criteria identified. After records were excluded, 833 articles were included and independently assessed for eligibility by JS, TS, SE and PS. A total of 38 articles were included at full-text screening, 17 removed at this stage, with 21 included in the meta-analyses (Figure 1).

### Characteristics of Studies

Five major outcomes of malnutrition were identified in the included studies: stunting (n=21), underweight (n=16), wasting (n=11), moderate or severe acute malnutrition (MAM/SAM) (n=1), and low birth weight (LBW) (n=4). Most studies utilised the WHO z-score to evaluate children's nutritional status. Seventeen studies examined maternal depression, while four assessed common mental disorders, employing either screening or diagnostic tools. The most frequently used validated tools were the Edinburgh Postnatal Depression Scale (EPDS), Self-Reporting Questionnaire (SRQ-20), Patient Health Questionnaire (PHQ-9), Centre for Epidemiologic Studies Depression Scale (CES-D), and the Structured Clinical Interview for DSM Disorders (SCID). Nonetheless, most studies used a screening tool to assess mothers' mental health. Three studies were conducted in a low-income country, Ethiopia. 13 studies were conducted in low-middle-income countries: 2 studies in each of Pakistan, Ghana, and Nepal; and 1 study in each of India, Bangladesh, Congo, Tanzania, Vietnam, Nigeria and Zimbabwe. The remaining five studies were conducted in upper-middle-income countries, including Brazil (n=2) and South Africa (3).

More than half of the included studies (12/21) were conducted in Sub-Saharan Africa. The country's classification is based on the classification provided by the World Bank [25]. For this study, low-middle-income countries and upper-middle-income countries are grouped as middle-income countries. The following Table 2 provides the summary of key data extracted from the included study.

Eight studies included in the meta-analysis were considered to have a low risk of bias (7 points or more on the NOS). 12 included studies were considered to have risk of bias (4-6 points on NOS). No included studies were considered to have very high risk of bias (0-3 points on NOS). Some of the common biases identified in the included studies were lack of representativeness of cases (hospital based), lack of clear control for key confounders, unclear or absent follow up duration. Full risk of bias assessments from all domains are included in Table 3 below.

### Meta-analysis results

#### Stunting (primary outcome)

Twenty-one studies were included in the meta-analysis of stunting as a binary outcome. Eight reported a significant increase in the level of stunting in children of mothers with depression or other mental health disorders. Nine reported a non-significant increase in the level of stunting in children of mothers with depression or other mental health disorders. Four studies reported a non-significant decrease in the level of stunting in children of mothers with depression or other mental health disorders.

A meta-analysis of the primary outcome revealed that a synthesised estimate of the adjusted odds ratio for the odds of stunted children of mothers with depression or other mental health disorders compared to the odds of stunted children of mothers with no depression or mental health disorders was 1.41 (95% CI 1.17, 1.69); i.e. odds of stunting were 41% higher in mothers with mental health issues. A *Z*-test of the standardised mean effect revealed strong evidence for a non-zero effect (*Z*=3.67; *p*<0.001). Individual estimates for the effect ranged from 3.34 (95% CI: 1.17, 9.51) [26] to 0.76 (95% CI: 0.37 to 1.53) [30]. A corresponding meta-analysis based on unadjusted estimates, conducted as a sensitivity analysis to assess the effect of the adjustments revealed a slightly higher synthesised estimate of 1.55 (95% CI 1.26 to 1.92), with no change to the inference of significance; suggesting that the variation in adjustments between studies had limited substantive effect.

Cochran’s χ^2^ test for heterogeneity revealed strong evidence for statistical heterogeneity (χ^2^_(20)_=59.2; *p*<0.001). The *I*^2^ statistic was revealed to be 66.2%, indicating a moderately high proportion of variation across studies ascribed to heterogeneity. The τ^2^ statistic (effect size variance) was revealed to be 0.103, corresponding to a 95% predictive interval for the effect (odds ratio) of (0.70, 2.83). The data is summarised in a forest plot (Figure 2).

A sensitivity analysis revealed no individual study to exert excessive influence on the analysis, with all point estimates of the omitted analyses lying within the 95% CI associated with the estimate of the combined analysis. Estimates and associated CIs are plotted on an influence plot (Figure 3).The contour-enhanced funnel plot for the stunting outcome (Figure 4), using the log of the odds ratio as the effect size, displayed possible evidence for small-study effects, with some included studies lying outside pseudo-95% confidence limits; and missing points representing smaller studies falling in both the significant and non-significant regions. As under publication bias, smaller studies are likely to be missing only in the non-significant regions, it is possible that the asymmetry observed is due to both publication bias and some other reason such as heterogeneity, which has already been established.

The Galbraith plot for the stunting proportion outcome revealed that 1 included study was outside the 95% CI region (given by the shaded area of the plot); a proportion approximately within expectations (Figure 5). The figure also illustrates high levels of precision of effects, with most studies being clustered away from the origin.

### Subgroup analyses

A subgroup analysis concerning stunting as a binary outcome conducted on studies assessed to be high quality (low risk of bias) using the NOS revealed no evidence for higher levels of stunting in children of mothers with depression or other mental health disorders. The odds ratio for stunting in children of mothers with depression or other mental health disorders compared to stunting in children of mothers without depression or other mental health disorders was 1.23; this represents a reduction from the value of 1.41 revealed in the corresponding analysis conducted on all included studies.

Subgroup analyses concerning stunting as a binary outcome conducted on *Age of child* revealed no evidence for higher levels of stunting in children aged up to 6 months of mothers with depression or other mental health disorders and evidence for higher levels of stunting in children aged up to 24 months of mothers with depression or other mental health disorder; compared to levels of stunting in children of mothers with no depression or other mental health disorder. As some studies featured in both subgroups, the between-groups effect was not calculated for these subgroups. Subgroup analyses with respect to the stunting proportion outcome conducted on *Country status* (comparing middle-income and low-income countries) revealed no evidence for higher levels of stunting in children of mothers with depression or other mental health disorders in low-income countries, and evidence for a difference in levels of stunting in children of mothers with depression or other mental health disorder in middle-income countries; compared to levels of stunting in children of mothers with no depression or other mental health disorder. The between-subgroup effect was revealed to be non-significant (Table 4).

### Secondary analyses

Meta-analyses conducted on the secondary outcomes revealed higher levels of standardised stunting in children of mothers with depression or mental health disorder compared with children of mothers with no depression or mental health disorder. No evidence for significant group differences was observed with respect to the underweight, wasting/moderate or severe malnutrition, WHZ and WAZ outcomes (Table 5).

## Discussion

This systematic review and meta-analysis examined the association between maternal mental health and child undernutrition from global literature. It provides comprehensive evidence supporting the association between maternal mental health disorder, and child undernutrition, specifically stunting and underweight, in children under five years of age. Our meta-analysis of the primary outcome indicated that maternal mental health disorders are significantly associated with increased odds of stunting in children, with 41% higher odds compared to children of mothers without mental health disorders (OR: 1.41; 95% CI, 1.17-1.69). No significant relationship was observed with wasting or underweight, even though increased odds of wasting and underweight were observed in children of mothers with mental health disorders. These lack of significant associations could be attributed to the acute nature of wasting and underweight, which are often influenced by episodic factors such as infections and short-term nutritional deficiencies [46,47]. The sensitivity analysis conducted using unadjusted estimates suggested 55% increased odds of stunting in mothers with mental health disorder, supporting the consistency in the relationship and robustness of the findings. While adjusted estimates are essential for controlling confounding factors and enhancing precision [48,49], the sensitivity analysis suggested that the observed association persists even in the presence of unadjusted confounders. However, when we restricted the sub-group analysis to high-quality studies (Low risk of bias) only, the odds ratio decreased to 1.23 and the association was no longer significant, possibly indicating the influence of methodological limitations in lower-quality studies on the overall analysis [50]. Although the odds ratio continued to suggest an increased risk, the attenuation observed in high quality studies indicates uncertainty regarding the true magnitude of association. Therefore, the magnitude of association should be interpreted with caution. These findings underscore the need for more high-quality studies with rigorous designs to clarify the effect size and strengthen the evidence base.

Further, subgroup analyses provided additional insights. We found that the odds ratio for stunting in children of mothers with mental health disorders was higher in LICs (OR: 1.90, 95% CI, 0.76-4.28) compared to MICs (OR:1.37, 95% CI, 1.14-1.66). However, the association was only statistically significant in MICs. This discrepancy in ORs and statistical significance could have resulted from methodological and contextual factors. Additionally, the lower number of studies from LICs, and the merging of low-middle-income and upper-income countries into a single MIC category in this study may have contributed to this discrepancy. Despite this, the higher odds ratio in LICs points to a possibly stronger link between child stunting and maternal mental health in these contexts. Elements like food insecurity, poverty, and limited access to healthcare may exacerbate the vulnerability of both mothers and children [15,46,51]. Nonetheless, these results suggest that maternal mental health plays a major contributing factor to child undernutrition. Therefore, it is necessary to conduct large-scale and reliable studies in LICs to provide accurate estimates and a deeper comprehension of the contextual factors that can influence the relationship between mothers' mental health and undernutrition in children.

Additionally, our sub-group analysis by children’s age showed a significant association between MMH disorders and stunting among children up to 24 months of age (12 studies; OR: 1.51, 95% CI: 1.11–2.04), whereas no statistically significant association was observed among infants younger than six months (3 studies; OR: 1.50, 95% CI: 0.79–2.87). Although significance was observed only in the older age group, the effect estimates were similar across both subgroups. The wider confidence interval and lack of statistical significance among infants under six months are likely attributable to the smaller number of studies and reduced statistical power rather than the absence of an association. Nevertheless, the findings suggest that MMH may influence child nutritional outcomes throughout early childhood. Given the critical role of maternal caregiving and feeding practices during this period, supporting MMH may contribute to improved child growth and development, particularly during the first two years of life [52-55]. Poor maternal mental health not only complicates adherence to nutritional recommendations, but can also adversely affect caregiving practices, and behaviours, such as feeding, responsiveness, and emotional availability, all of which can lead to poor nutritional outcomes for children [20,44,45,46,52]. Additionally, it is also important to recognize that the association between maternal mental health and undernutrition in children is complex and could be bidirectional. Poor nutritional status of children may exacerbate mental health issues in mothers by raising caregiver stress, anxiety and feelings of inadequacy [19,42,56]. On the other hand, maternal mental health can influence child nutrition [46,57]. Food insecurity may further reinforce this cycle, contributing to adverse outcomes for both mothers and children [58,59]. These findings highlight the complex interplay between MMH and child undernutrition, suggesting that interventions targeting only one component may have limited effectiveness.

Furthermore, since the goal of this review was to adopt a global perspective, it is noteworthy that studies from high-income countries (HICs) were not included in this analysis. This may reflect the lower prevalence of stunting and other traditional indicators of undernutrition in these settings. HICs have higher rates of obesity, micronutrient deficiencies, and non-communicable diseases linked to nutrition [60-62]. Future studies from HICs should examine whether MMH is associated with alternative nutrition-related outcomes, including obesity and micronutrient deficiencies. To provide a thorough understanding of MMH and child nutrition in various contexts, future research should concentrate on filling in these gaps. Different healthcare systems, cultural perspectives on mental health, and economic circumstances may all be responsible for these discrepancies [44,63]. The significance of context-specific interventions to address MMH and child nutrition in various socioeconomic contexts is highlighted by these findings. Effective results depend on interventions being specifically designed to address issues unique to a given region.

### Strengths and limitations

Our systematic review provides robust evidence on the association of maternal mental health disorders and undernutrition in children, building on previous systematic reviews in this field that have primarily focused on maternal depression and its association with child nutrition [11,20]. This review provides strong background evidence regarding the influence of all forms of mothers’ mental illness on child nutrition. While the study aimed to encompass broader aspects of maternal mental health, such as anxiety and common mental disorders, most studies examined depression specifically. Future research should further explore other dimensions of maternal mental health to build a more comprehensive understanding of its impact on child health outcomes.

The key strengths of this systematic review included the use of a comprehensive search to identify and select studies used in this review, hence minimising selection bias; and the inclusion of studies from across the globe assessing all forms of mental illness in mothers (depression, anxiety, common mental disorders) and undernutrition in children (stunting, wasting, underweight, MAM/SAM, LBW). Moreover, the use of the Newcastle-Ottawa Scale (NOS) which was used to assess the study’s quality, categorized none of the studies at high risk of bias, enhancing the confidence in findings and reducing the likelihood of bias affecting the pooled estimates.

Despite these strengths, certain limitations warrant consideration in our review. First, the high numbers of cross-sectional studies limit our ability to establish causality. Longitudinal studies are needed to explore these relationships more comprehensively and the dynamic interplay between maternal mental health and child nutritional outcomes over time. Studies assessing nutritional outcomes using micro and macro nutrients or obesity were not included in this study. Secondly, many articles in our review used screening tools to assess maternal mental health, it is important to note that they don’t provide a definitive diagnosis. Additionally, the cultural sensitivity or appropriateness of these tools in different context remain uncertain.

Only a few studies reported data by participant type and the intended subgroup analysis based on the sex of the child could not be conducted. Further, the between-groups effect for subgroups based on child age could not be derived due to a lack of data segregation. Findings from the subgroup analysis based on country income status may be limited by the disparity in the number of studies from different types of countries.

Finally, the observed statistical heterogeneity reflects the recognized clinical and methodological diversity across studies. Future research could further explore these variations to provide a more nuanced understanding of the relationship between maternal mental health and malnutrition outcomes.

## Conclusion

The findings of this meta-analysis indicate a significant association between maternal mental illness and child undernutrition; however, the attenuation observed in higher quality studies indicates uncertainty in the magnitude of this effect. Nonetheless, maternal mental health is intricately linked to child development; improving maternal mental health could contribute to better nutritional outcomes for children. Any intervention must take into account the socio-cultural factors specific to different countries and regions to ensure the effectiveness of such programs.

However, before designing tailored interventions, more context-specific research is required to understand the unique challenges faced in different settings. This includes further exploration of the mechanisms that drive the relationship between maternal mental health and child nutrition. Longitudinal studies are particularly important in identifying causal pathways and exploring how factors such as poverty, access to healthcare, and caregiving practices influence both maternal mental health and child nutritional outcomes. Policymakers should prioritise generating nationally representative data, particularly in LMICs, to inform effective public health strategies that address maternal mental health while improving child nutrition outcomes.

Integrated and cross-sectoral approaches are crucial for achieving meaningful progress. Aligning mental health services with nutritional support can address the interconnected factors affecting mothers and children. Programs targeting maternal mental health during critical periods, such as the first two years of a child’s life, may yield substantial benefits. Simultaneously, addressing food insecurity and strengthening social support systems should be integral to such interventions.

The absence of studies from high-income countries (HICs) highlights a gap in understanding maternal mental health and child nutrition in diverse settings. Stunting, the primary focus of this review, is less prevalent in HICs due to improved socioeconomic conditions and healthcare access [60]. Future research in HICs should explore how maternal mental health intersects with nutrition-related challenges prevalent in these contexts, such as obesity and micronutrient deficiencies. Expanding research to include HICs will provide a more comprehensive understanding of maternal mental health and its implications for child nutrition worldwide.

In conclusion, this review highlights the significant association between maternal mental health and child undernutrition, emphasizing the need for targeted interventions that address both maternal and child health. Contextual factors, such as economic conditions, cultural practices, and healthcare access, play a vital role in shaping this relationship. Future interventions should be informed by research that explores these dynamics, particularly in LMICs where the burden of maternal and child health challenges is greatest. By adopting integrated strategies that align maternal mental health and child nutrition programs, public health initiatives can more effectively address the complex, interrelated factors influencing the well-being of mothers and children.

## Figures and Tables

**Figure 1:**
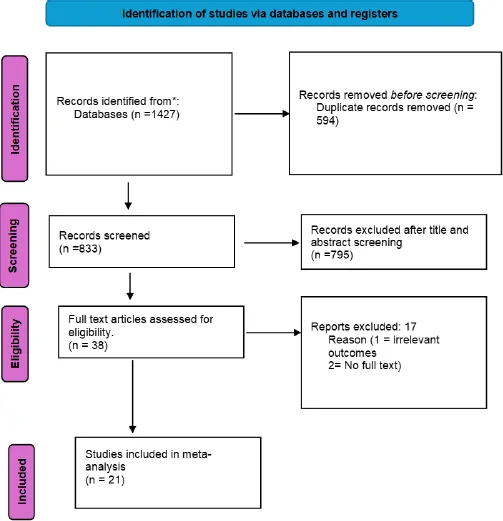
PRISMA flowchart

**Figure 2:**
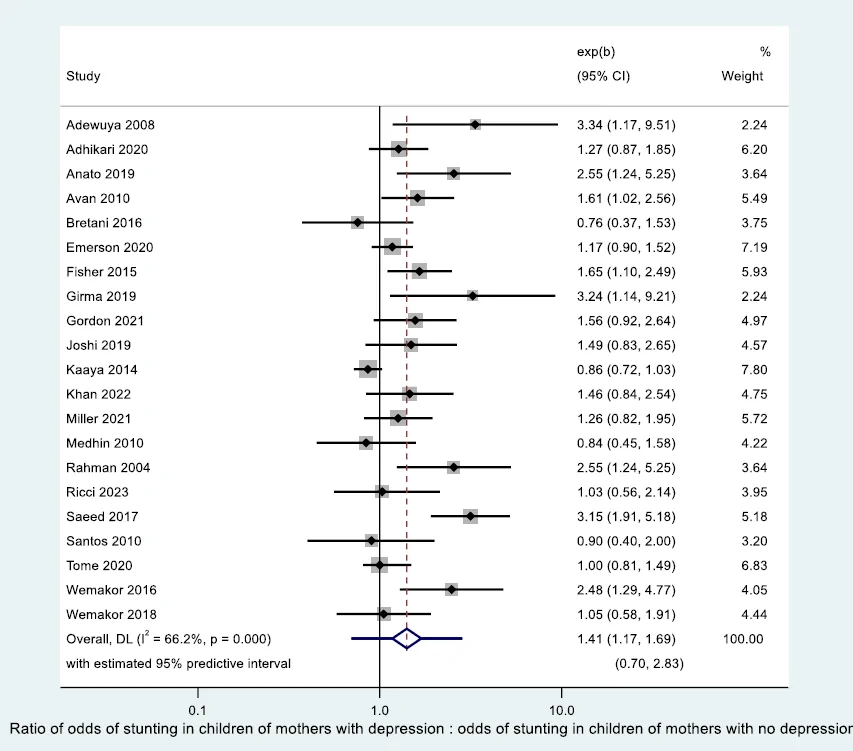
Forest plot for meta-analysis of stunting as a binary outcome

**Figure 3:**
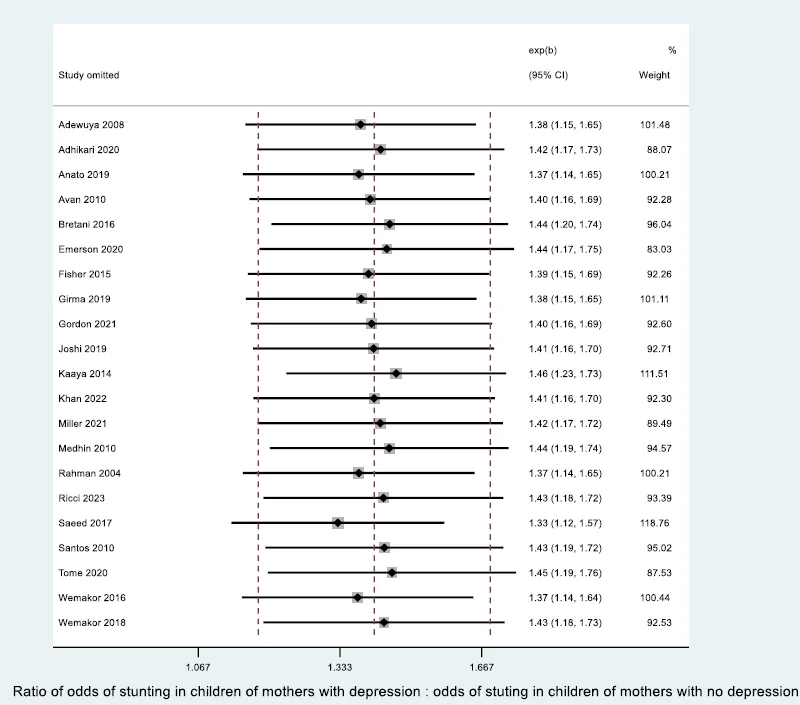
Influence plot for meta-analysis of stunting as a binary outcome

**Figure 4:**
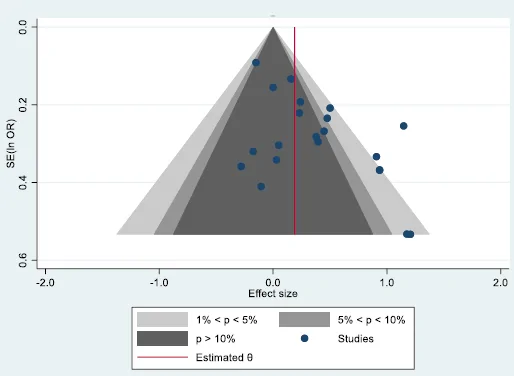
Contour-enhanced funnel plot for meta-analysis of stunting (as a binary outcome) with pseudo-95% confidence limits

**Figure 5:**
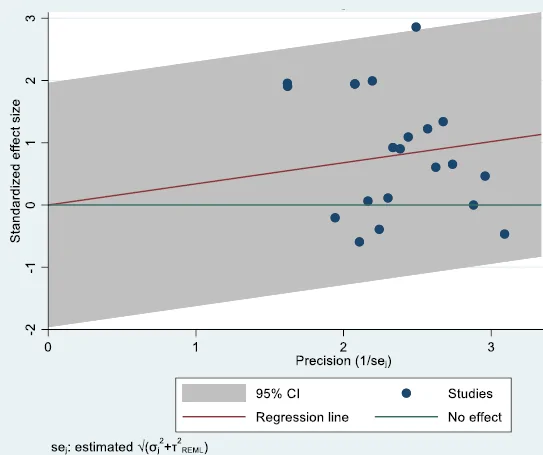
Galbraith plot for meta-analysis of stunting (as a binary outcome) with 95% confidence limits

**Table 1:** PECO summary table

Population	Exposure	Comparator	Outcome
**Children under 5 years of age, and their mothers.**	Mothers with mental disorders.	Mothers with no mental health disorders.	**Primary outcome:** unstandardised stunting **Secondary outcome:** Standardised stunting, standardised and unstandardised wasting or moderate or severe malnutrition; standardised and unstandardised underweight.

**Table 2:** Details of studies included in the review

Study	Design	Sample size	Children’s age group	Country	MMH assessment tool	Malnutrition assessment tool	MMH measurement	MMH prevalence	Stunting	Other forms of undernutrition
**Adewuya et al., 2008**	Longitudinal case control	242 (120 depressed, 122 matched non depressed)	Infants (first 9 months)	Nigeria	SCID-NP	WHO Weight and length measured against 5th percentile	Depression	NA, the study recruited 12- depressed vs 122 non-depressed mothers	Infants of depressed mothers were significantly more likely to have weight and length below the 5th percentile at 3 and 6 months.	NA
**Adhikari et al., 2020.**	Cross-sectional study	3158 mother-child, 826 fathers	6-59 months	Nepal	PHQ-9	WHO z-score	Depression	5%	28%	Underweight= 23%, Wasting= 10.6%
**Anato et al.,2020.**	Cross-sectional study	232 mother-infant	Infants (5-10 months)	Ethiopia	EPDS ≥ 13	WHO z score	Depression	22.80%	30.60%	Underweight= 7.8%, wasting= 20.7%
**Avan et al.,2010**	Longitudinal birth cohort	1035- Mothers, 651- children	Children at 2 years of age	South Africa	Pitt Inventory	WHO z-score	Depression	24%	16%	Underweight=3%, Wasting= 4%, LBW= 10.4%
**Brentani & Fink, 2016**	Cohort	798 mother-child dyads	Children below two years	Brazil	EPDS >13	Ages and stages Questionnaire (ASQ) WHO z- score	Depression	8.8%	7.3%	NA
**Emerson et al., 2020**	Cross-sectional	812 mother-child pair	6-59 months	Congo	Hopkins Symptom Checklist (HSCL-25)	WHO z- score	Depression	16.3%	63.8%	Underweight= 22.3% Wasting= 0.7%
**Fisher et al.,2015**	Population based prospective cohort study	S1-234 women AND S-2, 211 mothers and children	15 months old toddlers	Vietnam	SCID-I	WHO z-score	Common Mental disorder (CMD)	33.6 at baseline-s1, and 18.5 at follow-up (S2)	15.60%	NA
**Girma et al., 2019**	Unmatched Case control study	234 children, 78 cases (stunted children) and 156 controls (children without stunting)	24-59 months	Ethiopia	SRQ-20	MUAC WHO z-score	Common Mental disorder (CMD)	Mothers of cases= 53.8%, Mothers of controls= 13.5%	Cases only (100% by study design)	NA
**Gordon et al., 2021**	Longitudinal interventional study	1111 mothers	up to five years	South Africa	EPDS	WHO z-score	Depression	Pregnancy=13% Early childhood= 26.1% Recurrent= 20.3%	HAZ mean=0.49	WAZ= 0.16
**Joshi & Raut, 2019**	Cross-sectional study	300 mother-infant	Infants (0-11 months)	India	EPDS≥10	WHO z-score/ Growth standards	Depression	19%	44.60%	Underweight =33.60%, Wasting= 22.6%, LBW=25.7%
**Kaaya et al., 2016**	Retrospective cohort study	699 mother child pair	Mother-infant	Tanzania	Hopkins Symptoms checklist (HSCL-25)	WHO z-score	Depression	36.3%-Postdelivery Predelivery- 38.2%	HAZ/Stunting= 48.5%	WAZ/Underweight= 43.2% WHZ/Wasting= 23.3%
**Khan, 2022**	Cross-sectional	264 pairs of mothers and children	Mothers- under five children	Bangladesh	SRQ-20	WHO z-score	Common Mental Disorder (CMD)	46.2%	Stunting= 44.3%	Underweight=33.7% Wasting= 18.2%
**Medhin et al.,2010.**	Prospective cohort	873- at 6 months and 926 at 12 months	Infants (6-12 months)	Ethiopia	SRQ-20≥6	WHO z-score	Common Mental Disorder (CMD)	Pregnancy- 12%, Post-natal= 5%	At 6 months- 26%. At 12 months- 48%	Underweight at 6 months= 24%, Underweight at 12 months =22%
**Miller et al., 2021**	Cross-sectional	629 children	23-66 months	Nepal	EPDS	WHO z-score	Depression	21%	32%	Wasted= 13% Underweight= 30%
**Rahman et al., 2004**	Prospective cohort	632 healthy women in third trimester, 160 infants of depressed mothers and 160 infants of psychologically well mothers	infants	Pakistan	SCAN	WHO z-score	Depression	Prenatal depression 25%	2 months- 6%, 6 months- 10%, 12 months- 24%	Underweight in 2 months- 5%, 6 months- 18%, 12 months- 29%
**Ricci et al., 2023**	Cohort (follow up)	428 mothers infant	6 to <9 months of age (Infants)	South Africa	EPDS (<10)	WHO z-score	Depression	28.5%	LAAZ/HAZ >-2 (N=109)	N= 45 (underweight WAZ<-2)
**Saeed et al., 2017**	Cross-sectional study	325 mothers and children	Child less than 24 months	Pakistan	Aga Khan University Anxiety and Depression Scale≥20	WHO child growth standard scale	Depression	40%	36.60%	Underweight= 35.4%
**Santos et al.,2010**	Birth cohort	48 months-3792 chi, and 3748 mothers	12-48 months	Brazil	EPDS≥13	WHO child growth standard scale	Depression	17.9% in 3478 mothers. 4.7% persistently depressed at 12, 24 and 48 months	3.60%	Underweight= 1.7%, Wasting = 0.6% LBW=8.9%
**Tome et al., 2021**	Longitudinal study	4073 infants and 4025 mothers	Mother-infant dyad	Zimbabwe	EPDS	WHO z-score	Depression	8.8%	33.4%	NA
**Wemakor and Iddrisu, 2018**	Population based cross sectional study	200 mother-child pair	6-23 months	Ghana	CES-D≥20	WHO child growth standard scale	Depression	33.50%	41%	NA
**Wemakor and Mensah, 2016**	**Cross-sectional study**	**384 mothers**	**0-5 years**	**Ghana**	**CES-D**	**WHO child growth standard scale**	**Depression**	**27.80%**	**16.10%**	**Underweight= 8.9%, Wasting= 3.4% and LBW= 20.6%**

Abbreviations: MMH = maternal mental health; EPDS = Edinburgh Postnatal Depression Scale; PHQ-9 = Patient Health Questionnaire-9; SCID = Structured Clinical Interview for DSM; SRQ-20 = Self-Reporting Questionnaire-20; WHO = World Health Organization; MUAC = Mid-Upper Arm Circumference; LBW = low birth weight.

**Table 3:** Risk of bias of included studies using Newcastle-Ottawa scale

Study	Points awarded				Low risk of bias
	Selection	Comparability	Outcomes	Total	
**Adewuya et al., 2008 [26]**	2	2	1	5	×
**Adhikari et al., 2020 [27]**	2	2	1	5	×
**Anato et al., 2019 [28]**	3	2	1	6	×
**Avan et al., 2010 [29]**	3	2	1	6	×
**Bretani & Fink, 2016 [30]**	3	1	2	6	×
**Emerson et al., 2020 [31]**	3	1	2	6	×
**Fisher et al., 2015 [1]**	4	2	2	8	✓
**Girma et al., 2019 [32]**	4	2	3	9	✓
**Gordon et al., 2021 [33]**	4	2	3	9	✓
**Joshi & Raut, 2019 [34]**	3	2	1	6	×
**Kaaya et al., 2016 [35]**	3	2	3	8	✓
**Khan, 2022 [36]**	3	2	1	6	×
**Miller et al., 2021 [37]**	3	2	1	6	×
**Medhin et al., 2010 [38]**	3	2	3	8	✓
**Rahman et al., 2004 [39]**	4	2	1	7	✓
**Ricci et al., 2023 [40]**	4	1	3	8	✓
**Saeed et al., 2017 [41]**	3	2	1	6	×
**Santos et al., 2010 [42]**	4	2	3	9	✓
**Tome et al., 2021 [43]**	3	2	3	8	✓
**Wemakor et al., 2016 [44]**	3	2	1	6	×
**Wemakor et al., 2018 [45]**	3	0	1	5	×

**Table 4:** Subgroup analyses of the effect of maternal depression on stunting; by age of child and country status

Subgroup	Estimate (odds ratio) (95% CI)	Heterogeneity	Z-test for effect
Low risk of bias studies	1.23 (0.94, 1.61)	I^2^=64.7%	Z=1.52; p=0.130
Age of child Up to 6 months (n=3) Up to 24 months (n=12)	1.50 (0.788, 2.87) 1.51 (1.11, 2.04)	I^2^=63.9% I^2^=78.5%	Z=1.24; p=0.216 Z=2.63; p=0.009
Between-groups effect		Not calculated	
Country status Middle-income (n=18) Low-income (n=3)	1.37 (1.14, 1.66) 1.80 (0.758, 4.28)	I^2^=66.2% I^2^=73.0%	Z=3.34; p<0.001 Z=1.33; p=0.183
Between-groups effect		Z=0.602; p=0.795	

**Table 5:** Meta-analysis of secondary outcomes: synthesised effects and heterogeneity statistics

Outcome	Estimate (95% CI)	Heterogeneity	Z-test for effect
Standardised stunting (n=7)	^1^-0.13 (-0.24, -0.012)	I^2^=63.6%	Z=2.16; p=0.031
Wasting or moderate/severe malnutrition (n=8)	^2^1.64 (0.85, 3.18)	I^2^=83.7%	Z=1.48; p=0.139
WHZ scores (n=6)	^1^0.083 (-0.076, 2.43)	I^2^=62.2%	Z=1.02; p=0.306
Underweight (n=5)	^2^1.77 (0.94, 3.33)	I^2^=70.3%	Z=1.78; p=0.076
WAZ scores (n=6)	^1^-0.025 (-0.091, 0.041)	I^2^=0.0%	Z=-0.749; p=0.454

*^1^Standardised mean difference for effect in mothers with diagnosed depression or mental health disorder*

*^2^Odds ratio for effect in mothers with diagnosed depression or mental health disorder compared with children of mothers with no diagnosed depression or mental health disorder compared with children of mothers with no diagnosed depression or mental health disorder*
